# Importance and Presence of High-Quality Evidence for Clinical Decisions in Neurosurgery: International Survey of Neurosurgeons

**DOI:** 10.2196/ijmr.9617

**Published:** 2018-10-12

**Authors:** Jill Martens, Guido de Jong, Maroeska Rovers, Gert Westert, Ronald Bartels

**Affiliations:** 1 Neurosurgical Center Nijmegen Department of Neurosurgery Radboud University Medical Center Nijmegen Netherlands

**Keywords:** evidence-based medicine, neurosurgery, levels of evidence

## Abstract

**Background:**

The publication rate of neurosurgical guidelines has increased tremendously over the past decade; however, only a small proportion of clinical decisions appear to be based on high-quality evidence.

**Objective:**

The aim was to evaluate the evidence available within neurosurgery and its value within clinical practice according to neurosurgeons.

**Methods:**

A Web-based survey was sent to 2552 neurosurgeons, who were members of the European Association of Neurosurgical Societies.

**Results:**

The response rate to the survey was 6.78% (173/2552). According to 48.6% (84/173) of the respondents, neurosurgery clinical practices are based on less evidence than other medical specialties and not enough high-quality evidence is available; however, 84.4% (146/173) of the respondents believed neurosurgery is amenable to evidence. Of the respondents, 59.0% (102/173) considered the neurosurgical guidelines in their hospital to be based on high-quality evidence, most of whom considered their own treatments to be based on high-quality (level I and/or level II) data (84.3%, 86/102; significantly more than for the neurosurgeons who did not consider the hospital guidelines to be based on high-quality evidence: 55%, 12/22; *P*<.001). Also, more neurosurgeons with formal training believed they could understand, criticize, and interpret statistical outcomes presented in journals than those without formal training (93%, 56/60 and 68%, 57/84 respectively; *P*<.001).

**Conclusions:**

According to the respondents, neurosurgery is based on high-quality evidence less often than other medical specialties. The results of the survey indicate that formal training in evidence-based medicine would enable neurosurgeons to better understand, criticize, and interpret statistical outcomes presented in journals.

## Introduction

Evidence-based neurosurgery is a paradigm of neurosurgical practice in which the best available evidence is consulted to establish the principles of diagnosis and treatment. These principles are applied considering the neurosurgeon’s training and experience, as well as being informed by the patient’s individual circumstances and preferences, to produce the best possible health outcomes [1]. Although evidence-based medicine (EBM) is the gold standard in medicine [1-3], it is estimated that only 10% to 25% of clinical decisions are based on high-quality evidence [4], defined as level I and level II evidence (see Table 1 for definitions) [5].

**Table 1:** Levels of evidence in neurosurgery.

Level of evidence	Studies
I	(1) Randomized controlled trial, (2) meta-analysis of randomized controlled trials with homogeneous results
II	(1) Prospective comparative study (therapeutic), (2) meta-analysis of level II studies or level I studies with inconsistent results
III	(1) Retrospective cohort study, (2) case-control study, (3) meta-analysis of level III studies
IV	(1) Case series
V	(1) Case report, (2) expert opinion, (3) personal observation

In 2011, a new rating system, the Grading of Recommendations Assessment, Development, and Evaluation (GRADE), was developed, which offers an outcome-centric system for rating the quality of evidence derived from different types of studies [6]. The GRADE guidelines enable the rating of the quality of evidence in systematic reviews and clinical guidelines, as well as a determination of the strength of recommendations made in these documents. To the best of our knowledge, neurosurgery is currently still using the levels of evidence more often than the GRADE guidelines.

Rothoerl et al [7]. and Yarascavitch et al [5] published investigations into the levels of evidence in the neurosurgical literature in 2003 and 2012, respectively. These studies assigned a level of evidence to all published clinical papers in three major neurosurgical journals for the years 1999 and 2009-2010, respectively, graded according to the study design shown in Table 1 [5,8,9]. The authors found that 22.8% and 10.3% of evidence was considered higher-level evidence (level I or level II). Level I evidence, from randomized controlled trials yielding homogeneous results, was only found in 3.8% and 2.1% of the papers evaluated in these two studies, respectively.

These studies suggest that surgeons are increasingly turning their backs on research. Further evidence indicates that compared with a decade or two ago, surgeons apply for and receive fewer grants, publish less, and—perhaps most perniciously—feel that research is not part of their role [10]. Involvement in research allows surgeons to develop rigor in their everyday work and to judge, maintain, and improve the quality of the work done by their peers.

The goal of this study is to investigate the opinion of neurosurgeons about the evidence available in neurosurgical practice and the extent to which this evidence is implemented in clinical practice.

## Methods

### Recruitment

Evaluation by an ethical committee was not necessary for this study. A survey was conducted among 2552 members of the European Association of Neurosurgical Societies (EANS). The survey asked the opinion of neurosurgeons regarding the levels of evidence generated in neurosurgical studies, their understanding of the levels of evidence, and to what extent neurosurgeons implement evidence in clinical practice. The survey was emailed directly to the members of the EANS by the society’s administrative personnel. Data were collected over a period of 3 weeks from the date of the first mailing. Two reminders, each 1 week apart, were sent to the cohort.

### Survey

The survey (Multimedia Appendix 1) was made using a Google Inc program (Google Forms) and consisted of 13 sections containing a total of 22 questions. Sections with multiple questions within the survey were randomized to minimize the influence of the sequence of questions on the answers. Levels of evidence were used instead of ratings determined using the GRADE recommendations because the levels are still more commonly used by neurosurgeons to the best of our knowledge. Participants were asked for their opinions on high-quality evidence, the usability of the results of different research methods in clinical practice, the amenability of neurosurgery to evidence, the quality of guidelines in their hospital and of the guidelines used by the neurosurgeons themselves, and the most important factors for choosing between treatments. The guidelines mentioned in the questionnaire were selected in a previous study [11], in which a PubMed search was used to identify the most recent guidelines available. These guidelines were then characterized by the strength of their evidence [11]. This search covered the Agency for Healthcare Research and Quality (AHRQ) National Guidelines Clearinghouse and included both European guidelines and American guidelines. The participants were also asked whether they had received formal training in EBM, such as EU-ebm or CEBM, and if they considered themselves capable of understanding, criticizing, and interpreting statistical outcomes in journals.

Most questions consisted of a five-item Likert scale, which was chosen because each item is of equal value, so the respondents were scored rather than the items. The Likert scale was also likely to yield highly reliable answers and is easy to read and complete [12]. Some questions, for example about formal training in EBM and the ability to understand, criticize, and interpret statistical outcomes, were asked with a choice of Likert scale answers to enable the neurosurgeons to “rate” their training or ability to understand outcomes. The remaining questions had binary answers or were choices between statements. Participation was voluntary and completely anonymous, and the purpose of the survey was explained to the participants.

### Statistical Analyses

IBM SPSS version 22 (Armonk, NY, USA) was used for the statistical analyses. For the continuous data, Student *t* tests were used, whereas chi-square tests were used to analyze categorical data. Some values, for example the number of years as a neurosurgeon, were categorized before being statistically examined. Comparisons were made between different groups of neurosurgeons. Multiple groups were formed based on the answers to the questions asked and their opinions on different aspects, and consisted of respondents responding with a positive answer (“strongly agree,” “agree,” or “yes”), a negative answer (“strongly disagree,” “disagree,” or “no”), or an indecisive answer (“indifferent”) to particular questions. Afterwards, comparisons were made between the answers and opinions of certain groups for different aspects of neurosurgery. Values are presented as a mean ±95% confidence interval (CI).

## Results

The response rate was 6.78% (177 respondents) of the 2552 EANS members surveyed. All completed surveys contained complete data and had no partial or missing responses. Four respondents were excluded: three were still residents and one response was sent twice. A final total of 173 completed surveys (6.78%) were analyzed.

### Respondent Statistics

Table 2 shows the demographics of the respondents. Their years of experience varied. Most respondents (98.3%, 170/173) were specialized in one or more subspecialties. Of these, 85.9% (146/170) were specialized in two or more subspecialties, with a mean of 3.2 per person (95% CI 2.97-3.43). A total of 57.2% (99/173) of the respondents had one or more academic qualifications, such as a professorship or a PhD. The majority of the respondents (79.2%, 137/173) worked in one of 29 European countries, mostly Germany (10.4%, 18/173), the Netherlands (7.5%, 13/173), Greece (6.4%, 11/173), or the United Kingdom (5.8%, 10/173).

**Table 2:** Demographics of the respondents (N=173).

Demographics		n (%)
**Years working as a neurosurgeon**		
	1-5	30 (17.3)
	5-10	46 (26.6)
	10-15	38 (22.0)
	15-20	13 (7.5)
	20-25	13 (7.5)
	25-30	16 (9.3)
	>30	17 (9.8)
**Academic qualifications^a^**		
	Yes^b^	99 (57.2)
	Professor	28 (23.0)
	PhD	67 (54.9)
	MSPH	2 (1.6)
	MPH	5 (4.1)
	Other	20 (16.4)
**Subspecialty**		
	Yes^c^	170 (98.3)
	Neurocritical care	62 (11.7)
	Cerebrovascular neurosurgery	70 (13.2)
	Neuroendovascular surgery	14 (2.6)
	Spinal neurosurgery	121 (22.9)
	Neurosurgical oncology	125 (23.6)
	Pediatric neurosurgery	57 (10.8)
	Peripheral nerve neurosurgery	35 (6.6)
	Stereotactic and functional neurosurgery	30 (5.7)
	Other	15 (2.8)

^a^PhD: Doctor of Philosophy; MPH: Master of Public Health; MSPH: Master of Science in Public Health.

^b^28.6% of the neurosurgeons who answered “yes” had more than one academic qualification, with a mean of 1.2 per person (95% CI 1.11-1.29).

^c^85.9% of the neurosurgeons who answered “yes” had more than one subspecialty, with a mean of 3.2 per person (95% CI 2.97-3.43).

The remaining 36 respondents (20.8%, 36/173) worked in one of 18 non-European countries, particularly in countries in the Middle East (11.6%, 20/173), such as Saudi Arabia (2.9%, 5/173), Pakistan (2.3%, 4/173), and Iraq (1.7%, 3/173). The remaining respondents came mainly from Mexico (1.7%, 3/173), India (1.7%, 3/173), and the United States (1.2%, 2/173).

### Evaluation Outcomes

**Figure 1:**
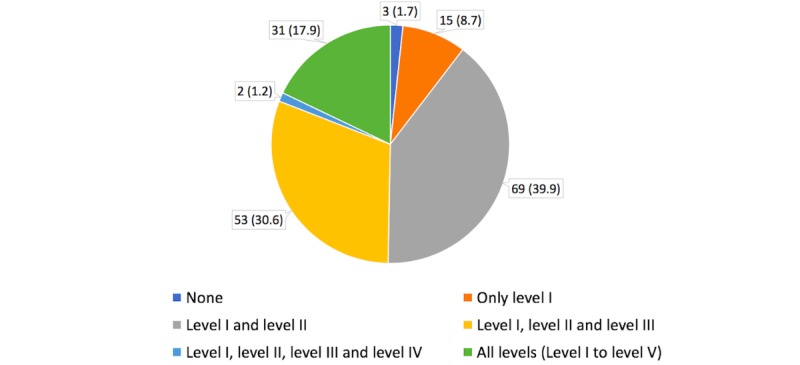
Levels of evidence used by neurosurgeons in clinical practice. The results are presented on a five-item Likert scale: (1) strongly agree, (2) agree, (3) indifferent, (4) disagree, or (5) strongly disagree.

Table 3 shows the opinions of the respondents regarding the levels of evidence and the use of EBM in clinical practice. Of the 173 respondents, 84 (48.6%) considered level I or level I and level II evidence to be of high quality. Figure 1 shows the levels of evidence used by neurosurgeons in clinical practice; most respondents implemented all levels of evidence into their clinical practice. The results of randomized controlled trials (RCTs) with inconsistent, but promising, results were used by fewer than half of the respondents (45.7%, 79/173; Tables 4 and 5).

Every participant indicated the guidelines they used most often, to a maximum of three guidelines (Table 6). The level of evidence generated by the research underpinning each guideline is shown. Guidelines considering the surgical management of traumatic brain injury were most commonly used (39.3%, 68/173), followed by those for severe traumatic brain injury (38.2%, 66/173) and subarachnoid hemorrhage (38.2%, 66/173). Table 6 also shows the number of neurosurgeons using the guideline who subspecialized in the corresponding area of neurosurgery. The numbers of neurosurgeons specializing in the areas corresponding to the three most-used guidelines did not comprise a large percentage of the total number of neurosurgeons using these guidelines (48.5%, 48.5%, and 78.8% of neurosurgeons specializing in the surgical management of traumatic brain injury, severe traumatic brain injury, and subarachnoid hemorrhage, respectively). This may be because these three areas are all critical conditions that require immediate care; therefore, it is likely that most neurosurgeons will use these guidelines even if it is not their subspecialty.

**Table 3:** Levels of evidence considered by neurosurgeons to be of high quality and usable in clinical practice.

Levels of evidence	Considered to be of high quality and usable in clinical practice, n (%)
None	3 (1.7)
Level I	15 (8.7)
Level I and level II	69 (39.9)
Level I, level II, and level III	53 (30.6)
Level I, level II, level III, and level IV	2 (1.2)
All levels (Level I-V)	31 (17.9)

**Table 4:** Studies used by the participants in clinical practice. Scores were given from 1 (strongly disagree) to 5 (strongly agree).

Level of evidence	Studies	Studies used, mean (95% CI)
I	(1) RCT^a^, (2) meta-analysis of RCTs with homogeneous results	3.8 (2.14-5.46)
II	(1) Prospective comparative study (therapeutic)	3.9 (2.48-5.32)
II	(2) Meta-analysis of level II studies	3.9 (2.34-5.46)
II	(3) Meta-analysis of level I studies with inconsistent results	3.3 (1.54-5.06)
III	(1) (Meta-analysis of) retrospective cohort study	3.8 (2.36-5.24)
III	(2) Case-control study	3.6 (2.06-5.14)
IV	(1) Case series	3.7 (2.02-5.38)
V	(1) Case report, (2) expert opinion, (3) personal observation	3.5 (1.64-5.36)

^a^RCT: randomized controlled trial.

**Table 5:** Summary of the overall survey results (N=173).

Survey item		Strongly agree or agree, n (%)	Indifferent, n (%)
**Factors important for choosing a treatment**			
	Clinical experience is an important factor for choosing a treatment	172 (99.4)	0 (0.0)
	Research is an important factor for choosing a treatment	160 (92.5)	10 (5.8)
	Knowledge from patients and carers is an important factor for choosing a treatment	124 (71.7)	39 (22.4)
	Local context and environment are important factors for choosing a treatment	123 (71.1)	42 (24.3)
**Use of research in clinical practice**			
	I use prospective cohort studies in clinical practice	134 (77.5)	34 (19.7)
	I use meta-analysis of prospective cohort studies in clinical practice	130 (75.1)	35 (20.2)
	I use (meta-analysis of) retrospective cohort studies in clinical practice	126 (72.9)	39 (22.5)
	I use (meta-analysis of) RCTs^a^ with homogeneous results in clinical practice	122 (70.5)	41 (23.7)
	I use case-control studies in clinical practice	113 (65.3)	42 (24.3)
	I use case series in clinical practice	111 (64.2)	47 (27.2)
	I use case reports, expert opinions, or personal observations in clinical practice	98 (56.6)	52 (30.1)
	I use (meta-analysis of) RCTs with inconsistent, but promising, results in clinical practice	79 (45.7)	66 (38.2)
**Guidelines and treatment options**			
	Treatment options I use are based on high-quality evidence	129 (74.5)	26 (15.0)
	The neurosurgeons at my hospital are involved in the process of setting up the neurosurgical guidelines for my hospital	126 (72.8)^b^	3 (1.7)^c^
	Guidelines at my hospital are based on high-quality evidence	102 (59.0)	49 (28.3)
**Training**			
	I can understand, criticize, and interpret statistical outcomes in journals	87 (80.3)	19 (11.1)
	I have received formal training in EBM^d^	60 (34.7)	29 (16.8)
Neurosurgery is amenable to evidence		146 (84.4)	19 (11.0)

^a^RCT: randomized controlled trial.

^b^Question was answered with “yes.”

^c^Question was answered with “other.”

^d^EBM: evidenced-based medicine.

**Table 6:** Most-used neurosurgical guidelines (N=173).

Guidelines			Level of evidence in research used to develop guideline [11]	Neurosurgeons using this guideline, n (%)		Neurosurgeons using this guideline subspecialized in this field, n (%)
**Head injury**			—^a^	150 (86.7)		—
	Surgical management of traumatic brain injury	Moderate		68 (39.3)	33 (49)	
	Severe traumatic brain injury	Moderate		66 (38.2)	32 (49)	
	Pediatric traumatic brain injury	Moderate		9 (5.2)	7 (78)	
	Mild traumatic brain injury	High/moderate		7 (4.0)	4 (44)	
**Spine**			—	136 (78.6)		—
	Lumbar disk herniation	All levels		38 (22.0)	32 (84)	
	Cervical spine and spinal cord injury	Moderate		19 (11.0)	19 (100)	
	Degenerative lumbar spondylolisthesis	Moderate/low		17 (9.8)	15 (88)	
	Degenerative lumbar stenosis	Moderate/low		13 (7.5)	10 (77)	
	Degenerative cervical spine disease	Moderate		11 (6.4)	11 (100)	
	Lumbar spine fusion	All levels		11 (6.4)	10 (91)	
	Cervical radiculopathy and degenerative disease	All levels		10 (5.8)	10 (100)	
	Antibiotic prophylaxis in spine surgery	All levels		9 (5.2)	7 (78)	
	Intraoperative spinal monitoring	High		6 (3.5)	4 (67)	
	Somatosensory evoked potentials	Moderate		1 (0.6)	1 (100)	
	Vertebral osteomyelitis, diskitis, and epidural abscess	Moderate/low		1 (0.6)	1 (100)	
**Vascular**			—	96 (55.5)		—
	Subarachnoid hemorrhage	All levels		66 (38.2)	52 (79)	
	Intracerebral hemorrhage	High		23 (13.3)	19 (83)	
	Extracranial carotid disease	High/moderate		7 (4.0)	6 (86)	
**Tumor**			—	81 (46.8)		—
	Glioblastoma	Moderate		60 (34.7)	52 (87)	
	Brain metastases	High/moderate		21 (12.1)	18 (86)	
**Functional**			—	11 (6.4)		—
	Deep brain stimulation	High/moderate		10 (5.8)	9 (90)	
	Vagal nerve stimulation	Moderate		1 (0.2)	0 (0)	
**Other**			—	22 (4.4)		—
	Hydrocephalus	Moderate		18 (3.6)	14 (78)	
	Carpal tunnel syndrome	High/moderate		4 (0.8)	1 (25)	

^a^Not applicable.

According to 84.4% of the neurosurgeons (146/173), neurosurgery is amenable to evidence (Table 5); however, nearly half of the respondents (48.6%, 84/173) believed that neurosurgery is less based on evidence than other medical specialties. Despite this, 74.6% of the respondents (129/173) consider their treatments to be based on level I and/or level II evidence. Of those who believed neurosurgery is amenable to evidence, 78.8% (115/146 respondents) considered their treatments to be based on level I and/or level II evidence, whereas significantly fewer (25%, 2/8) of those who did not believe neurosurgery is amenable to evidence considered their research to be based on such high-quality evidence (*P*=.048). Of the most-used neurosurgical guidelines (Table 6), only 9.78% (242/2469) were based on level I evidence, whereas 20.43% (545/2668) were based on level II evidence.

Of the 129 respondents who believed their treatments were based on high-quality evidence, 50.4% (65/129) considered level I and/or level II to be high-quality evidence, whereas significantly fewer (39%, 7/18) of the respondents who did not consider their treatments to be based on high-quality evidence considered level I and/or level II to be high quality (*P*=.02).

Most respondents (72.8%,126/173) were involved in the process of setting up the neurosurgical guidelines in their hospital. More than half (59.0%, 102/173) of the respondents considered the neurosurgical guidelines of their hospital to be based on high-quality evidence. Of those who were involved in their establishment, 65.9% (83/126) considered the guidelines of their hospital to be based on high-quality evidence, compared with just 43% (20/47) of those who were not involved (*P*=.02).

Of the 59.0% (102/173) of respondents who considered the neurosurgical guidelines in their hospital to be based on high-quality evidence, 84.3% (86/102) considered their own treatments to be based on level I and/or level II evidence, whereas 55% (12/22) of the respondents who did not consider hospital guidelines to be based on high-quality evidence considered their own treatments to be based on level I and/or level II evidence (*P*<.001).

Only 34.7% (60/173) of the respondents said they had received formal training in EBM. Of those who received formal training, 76% (46/60) considered their own treatments to be based on level I and/or level II evidence, whereas 85% (71/84) of those without formal training believed their treatments were based on this level of evidence (*P*=.03).

The majority of respondents (80.3%, 139/173) said they could understand, criticize, and interpret statistical outcomes in medical research. This response was more common for respondents who received formal training (93%, 56/60) than for those without formal training (68%, 57/84; *P*<.001). There was no difference between the number of respondents with and without additional academic qualifications who stated that they could understand, criticize, and interpret statistical outcomes (85%, 84/99 and 74%, 55/74, respectively; *P*=.16).

All participants had the option to add their own comments at the end of the survey. The most frequent comment was that the lack of evidence is an important issue in neurosurgery. Neurosurgeons also said that RCTs are expensive and difficult to perform, although well-designed prospective comparative studies could be equally informative and easier to run. They therefore concluded that dismissing study designs other than RCTs when developing neurosurgical guidelines is holding back neurosurgery.

## Discussion

This study is unique because it is, to the best of our knowledge, the first to evaluate the opinion of neurosurgeons in several countries regarding the use of evidence in neurosurgery. Level I and level II evidence is considered high quality; however, despite a worldwide acceptance of this classification, only 48.5% of the respondents (84/173) considered either level I or levels I and II to be high quality. Moreover, all levels of evidence seem to be used by the majority of neurosurgeons. Several neurosurgeons commented that the lack of evidence is an important issue in neurosurgery.

Indisputable advancements in neurosurgery have been traditionally based on technical innovations advocated by pioneers without rigid assessment in clinical trials; therefore, changes in clinical practice have frequently been technology-driven rather than strictly evidence-based [13]. Everyday clinical management in neurosurgery does not, therefore, always seem to comply with the best available evidence.

Since 1970, the rate of increase in the publication of guidelines in all specialties has outpaced neurosurgery [11]; however, in the past 5 years, the number of guidelines published per year in neurosurgery has increased at the same rate as all specialties [11]. The available literature shows that neurosurgery uses a higher percentage of high-level evidence than some other specialties, including general plastic surgery [14] and maxillofacial surgery [15]; however, neurosurgery is still lagging behind many other specialties, including orthopedics [16], ophthalmology [17], otolaryngology [18], esthetic surgery [19], and urology [20]. The situation in other fields resembles that of neurosurgery; for example, 12.2% of the treatment for atrial fibrillation is based on level I and II evidence [21], although we could not find any data on the levels of evidence used in the treatment of cardiovascular disease as a whole. When comparing neurosurgery with oncology, we discovered that oncology uses the AGREE (Appraisal of Guidelines for Research and Evaluation) rating of guidelines [22-25]. The AGREE domains (scope and purpose, stakeholder involvement, rigor of development, clarity of presentation, applicability, and editorial independence) [26] are not comparable with the levels of evidence, in which studies are graded by study design.

Of the respondents who participated in this study, 25.4% (44/173) did not think that, or know whether, the treatment options they use are based on high-quality evidence. Ducis et al [11] investigated the quality of the guidelines used in neurosurgery clinical practice. In neurosurgery, 24.4% of the guidelines were based mainly on level I recommendations, whereas for vascular neurosurgery guidelines this percentage is significantly higher: 51.9%. Some other specialties have numbers of level I-based recommendations similar to neurosurgery, including endocrinology [27], infectious diseases [28], and hepatology [29]. Vascular neurosurgery is the subspecialty with the highest publication rate in neurosurgery [5], and vascular neurosurgery guidelines are the third most commonly used in clinical practice (according to Table 6). Guidelines relating to traumatic brain injuries are the most used according to the respondents, but this subspecialty accounted for just 6.4% of neurosurgery publications between 2009 and 2010 [5]. The level I-based recommendations for traumatic brain injury guidelines accounted for only 5.6% of all recommendations [11], significantly less than the level I-based recommendations for spine guidelines (10.0%) and vascular guidelines (51.0%), as assessed with a chi-square test (*P*<.001).

One participant commented that neurosurgery is currently based more on eminence than on evidence. Eminence refers to a clinical decision that is made solely by relying on the opinion of a medical specialist or any prominent health professional rather than the critical appraisal of the scientific evidence available [30]. Evidence is an integration of clinical knowledge and skills with the best critically appraised research available, as well as patient values and preferences, in order to make a clinical decision [30]. With the lack of evidence available to neurosurgeons, neurosurgery seems indeed to be based more on eminence than evidence in some cases.

Another neurosurgeon commented that the current definition of evidence-based neurosurgery is in dire need of rigorous update and expansion. A common misunderstanding of EBM is that a lack of available evidence means a lack of RCTs [31]. EBM evaluates the quality of evidence, based primarily on the likelihood that the evidence is biased. A powerful RCT is the best standard for evaluating this inherent bias, but it does not follow that only RCTs can be used to justify clinical practice in EBM; rather, EBM requires that we attempt to audit our decisions by obtaining the highest level of evidence ethically or logistically possible [31,32].

The differences in the confidence of neurosurgeons with and without formal training in EBM to adequately interpret the statistical outcomes presented in the literature was striking. Only 35% of the responding neurosurgeons (60/173) had received formal training in EBM and although it seems low, it is similar to that described in orthopedic surgery [33]. The lack of EBM training in neurosurgery has already been noted elsewhere; the University of Western Ontario in London, ON, Canada [34], and the American Accreditation Council for Graduate Medical Education [31] recently incorporated EBM into the curriculum for residency training programs in neurosurgery. Since EBM is based on the implementation of correctly interpreted research results, our findings may represent an argument for the introduction of more formal EBM training in the medical curriculum and supplementary training for neurosurgical departments.

A major strength of this study is the broad representation of the opinions of neurosurgeons worldwide through the involvement of the EANS. EANS has a large number of members all over the world. The membership of EANS is primarily located in Europe, but neurosurgeons from all countries are permitted to join. However, some potential limitations should also be discussed. First, due to the low response rate, selection bias cannot be precluded, which hampers the generalizability of our results. For external email surveys, a response rate between 10% to 25% is usually considered the average [35,36]. The response rate to our survey was a little lower than average, which might be caused by a general lack of interest by neurosurgeons or because they did not recognize the importance of this study. Bias could have been introduced because the survey may have been selectively answered by those who consider EBM important, specifically by those who were trained in EBM, involved in innovation and guidelines, or opinion leaders. Second, the participants of the survey may have chosen to provide socially desirable answers; however, this was counteracted by emphasizing the anonymity of the survey. A third potential issue is that neurosurgery is in the middle of transitioning from rating the guidelines using levels of evidence to rating them with the GRADE guidelines. We choose to use the levels of evidence here because they are currently more widely used by neurosurgeons; however, this could have been confusing for those who have already begun to use the GRADE guidelines.

The transition from using levels of evidence to the GRADE system is an important change in all medical specialties, including neurosurgery. The literature available about the evaluation of evidence in neurosurgery does not include the new system and is, therefore, in need of expansion and updating. The GRADE guidelines offer some major advantages over the levels of evidence, as they enable the rating of evidence in systematic reviews and guidelines and the grading of the strength of recommendations made in guidelines. Moreover, the GRADE system offers a transparent and structured process for developing and presenting summaries of evidence in systematic reviews and guidelines in health care, and for carrying out the steps involved in developing recommendations [6].

There are major gaps between the current definition of high-quality evidence (level I and level II) and neurosurgeons’ opinions of evidence. Neurosurgeons are willing to base their clinical decisions on more than just RCTs, relying on lower-quality evidence. The transition to the GRADE system is one way to overcome this issue because these guidelines are outcome-centric and do not rate each study as a single unit. In the GRADE approach, RCTs are initially considered to be high-quality evidence and observational studies are initially considered low-quality evidence supporting estimates of intervention effects. Five factors may lead the ratings to be decreased, whereas three other factors may lead to an increased rating. Ultimately, the quality of evidence for each outcome falls into one of four categories, from high to very low [6].

EBM is important when choosing between treatments for patients; however, shared decision making (SDM) was also developed alongside the introduction of EBM. According to 71.7% (124/173) of the respondents of this study, knowledge gained from patients and carers was an important factor in the selection of a treatment, whereas 22.5% of the respondents (39/173) were indifferent and 5.8% (10/173) did not believe that the knowledge of patients and carers was an important factor when choosing a treatment. Evidence from trials has shown that engaged patients consume less health care resources [37,38]; furthermore, when doctors are too focused on EBM, preference misdiagnoses (also known as silent misdiagnoses) can be made, causing the patient to receive an unwanted treatment [39]. EBM is important but has to be combined with SDM to give patients the right treatment. Learning to combine these two sides of medicine could be an important development in all specialties, leading to the optimization of health care for patients.

Understanding EBM is key to using it correctly in practice. This study shows that relatively few neurosurgeons have received formal training in EBM; thus, more training in EBM, both in the medical curriculum for residents and at neurosurgical departments, would enable neurosurgeons to improve their abilities to better facilitate the implementation of statistical results into clinical practice. It would be interesting to perform this research on a larger scale, including a wider multinational sample in the future.

According to the respondents, neurosurgery is less commonly based on high-quality evidence than other medical specialties. The results of the survey suggest that providing more formal training in EBM is desirable, enabling neurosurgeons to better understand, criticize, and interpret the statistical outcomes presented in journals.

## Appendix

Multimedia Appendix 1 "Evidence in Neurosurgery" survey.

## Abbreviations

AGREE: Appraisal of Guidelines for Research and Evaluation
AHRQ: Agency for Healthcare Research and Quality
EANS: European Association of Neurosurgical Societies
EBM: evidence-based medicine
GRADE: Grading of Recommendations Assessment, Development, and Evaluation
RCT: randomized controlled trial
SDM: shared decision making
