# Supplementary material for: Importance and Presence of High-Quality Evidence for Clinical Decisions in Neurosurgery: International Survey of Neurosurgeons
Source: Interact J Med Res. 2018 Oct 12;7(2):e16. doi: 10.2196/ijmr.9617 (PMC6231869; doi:10.2196/ijmr.9617)
Supplement: Multimedia Appendix 1 [file ijmr_v7i2e16_app1.pdf]

Multimedia Appendix 1: Survey 'Evidence in Neurosurgery'

Survey 'Evidence in Neurosurgery'

This survey is about the opinion of neurosurgeons regarding evidence in neurosurgery. The survey will take you approximately 10 to 15 minutes and is completely anonymous.
Thank you in advance for answering.

Section 1 of 13

What country do you work in?

____

Section 2 of 13

How many years have you been working as a neurosurgeon?

____

Section 3 of 13

What are the subspecialties you operate in?

- No subspecialisation
- Neurocritical care
- Cerebrovascular neurosurgery
- Neuroendovascular surgery
- Spinal neurosurgery
- Neurosurgical oncology
- Pediatric neurosurgery
- Peripheral neurosurgery
- Stereotactic and functional neurosurgery
- Other: ____

Section 4 of 13

Do you have additional academic qualifications?

- No
- PhD
- MPH (Master of Public Health)
- MSPH (Master of Science in Public Health
- Professor
- Other: ____

Section 5 of 13

Are the neurosurgeons at your hospital involved in the process of setting up the neurosurgical guidelines?

- Yes
- No
- Other: ____

Section 6 of 13

The following factors are important for choosing a treatment.

	Strongly agree	Agree	Indifferent	Disagree	Strongly disagree
Research					
Clinical experience					
Knowledge from patients and carers					
Local context and environment					

Section 7 of 13

Which level(s) of evidence do you consider high-quality and use in clinical practice?

- None
- Only Level I
- Level I and Level II
- Level I, Level II and Level III
- Level I, Level II, Level III and Level IV
- All Levels (Level I to Level V)

Section 8 of 13

The neurosurgical treatment options I use are based on Level I and/or Level II evidence.

- Strongly agree
- Agree
- Indifferent
- Disagree
- Strongly disagree

Choose a maximum of three treatment guidelines you use most.

- HEAD INJURY: Severe traumatic brain injury
- HEAD INJURY: Surgical management of traumatic brain injury
- HEAD INJURY: Pediatric traumatic brain injury
- HEAD INJURY: Mild traumatic brain injury
- SPINE: Degenerative lumbar spondylolisthesis
- SPINE: Lumbar spine fusion
- SPINE: Lumbar disk herniation
- SPINE: Degenerative lumbar stenosis
- SPINE: Cervical spine and spinal cord injury
- SPINE: Degenerative cervical spine disease
- SPINE: Cervical radiculopathy and degenerative disease
- SPINE: Antibiotic prophylaxis in spine injury
- SPINE: Vertebral osteomyelitis, diskitis, and epidural abscess
- SPINE: Somatosensory evoked potentials
- SPINE: Intraoperative spinal monitoring
- VASCULAR: Subarachnoid hemorrhage
- VASCULAR: Intracerebral hemorrhage
- VASCULAR: Venous sinus thrombosis
- VASCULAR: Extracranial carotid disease

TUMOR: Brain metastases
TUMOR: Glioblastoma
FUNCTIONAL: Vagal nerve stimulation
FUNCTIONAL: Deep brain stimulation
OTHER: Carpal tunnel syndrome
OTHER: Hydrocephalus
Other: ____________________________________

Section 9 of 13

Neurosurgery is amenable to evidence. (It is not only clinical experience.)
Strongly agree
Agree
Indifferent
Disagree
Strongly disagree

Choose the statement you agree most with.
Neurosurgical practice is based on more evidence compared with other medical specialties.
Neurosurgical practice is equally based on evidence compared with other medical specialties.
Neurosurgical practise is based on less evidence compared with other medical specialties.

Section 10 of 13
The following questions will give you statements about researches. Consider if you use the given research in your clinical approach for treating a patient.

I use RCTs or meta-analysis of RCTs with homogeneous results in clinical practice.
Strongly agree
Agree
Indifferent
Disagree
Strongly disagree

I use a proper meta-analysis of RCTs with promising, but inconsistent results, in clinical practice.
Strongly agree
Agree
Indifferent
Disagree
Strongly disagree

I use a proper meta-analysis of prospective comparative studies in clinical practice.
Strongly agree
Agree
Indifferent
Disagree
Strongly disagree

I use prospective comparative studies in clinical practice.
Strongly agree
Agree
Indifferent
Disagree
Strongly disagree

I use a proper retrospective cohort study or meta-analysis of retrospective cohort studies in clinical practice.
Strongly agree
Agree
Indifferent
Disagree
Strongly disagree

I use a proper case-control study in clinical practice.
Strongly agree
Agree
Indifferent
Disagree
Strongly disagree

I use a proper case series in clinical practice.
Strongly agree
Agree
Indifferent
Disagree
Strongly disagree

I use a case report, expert opinion or personal observation in clinical practice.
Strongly agree
Agree
Indifferent
Disagree
Strongly disagree

Section 11 of 13

The neurosurgical guidelines in my hospital are based on high-quality evidence.
Strongly agree
Agree
Indifferent
Disagree
Strongly disagree

Section 12 of 13

I receive formal training in evidence-based medicine (such as EU-ebm, CEBM or online courses).

- Strongly agree
- Agree
- Indifferent
- Disagree
- Strongly disagree

I can understand, criticize and interpret statistical outcomes presented in journals. (For example, P-value.)

- Strongly agree
- Agree
- Indifferent
- Disagree
- Strongly disagree

Section 13 of 13

Comments (optional)

____
